# *Actinospica acidithermotolerans* sp. nov., a novel actinomycete isolated from sediment from an Indonesian hot spring

**DOI:** 10.1007/s00203-022-03058-7

**Published:** 2022-07-23

**Authors:** Ali B. Kusuma, Kurniawan E. Putra, Leggina R. Vanggy, Joshua Loh, Imen Nouioui, Michael Goodfellow

**Affiliations:** 1 School of Natural and Environmental Sciences, Ridley Building, Newcastle University, Newcastle upon Tyne, NE1 7RU UK; 2 Indonesian Centre for Extremophile Bioresources and Biotechnology (ICEBB), Faculty of Life Sciences and Technology, Sumbawa University of Technology, Sumbawa Besar, 84371 Indonesia; 3 Leibniz-Institut DSMZ-German Collection of Microorganisms and Cell Cultures, Inhoffenstraße 7B, 38124 Braunschweig, Germany; 4 Northumbria University, Newcastle upon Tyne, NE1 8ST UK; 5 Department of Research, Innovation and Development, Sumbawa Technopark (STP), Sumbawa Besar, 84371 Indonesia

**Keywords:** *Actinospica acidithermotolerans*, Acidophilic actinomycetes, Polyphasic taxonomy, Genomics, Genome mining

## Abstract

A polyphasic study was designed to resolve the taxonomic position of isolate MGRD01-02^T^ which was recovered from an acidic hot spring in Indonesia and assigned to the genus *Actinospica*. Phylogenetic analyses based on 16S rRNA gene sequences show that the isolate is most closely related to the type strains of *Actinospica acidiphila* (98.5%), *Actinospica robiniae* (97.8%) and *Actinospica durhamensis* (96.8%). Morphological and chemotaxonomic data underpin the assignment of the isolate to the genus *Actinospica* as it forms an extensively branched substrate mycelium which carries tufts of white aerial hyphae that differentiate into straight to flexuous chains of cylindrical spores with faint rugose surfaces, contains 2,6-diamino-3-hydroxydiaminopimelic acid in the peptidoglycan, mixtures of hydrogenated menaquinones with nine isoprene units, *iso-*C _15:O_ and *iso*-C _16:O_ as major fatty acids and phosphatidylethanolamine as the diagnostic phospholipid. Whole-genome sequence analyses show that the isolate, *A. durhamensis* CSCA 57^T^ and *Actinocrinis puniceicyclus* DSM 45168^T^ have genome sizes of 7.9, 9.6 and 6.7 Mbp, respectively. A phylogenomic tree shows that they form distinct branches in a well-supported clade, a result supported by associated phenotypic data. Average nucleotide identity and digital DNA:DNA hybridization similarities are below the recommended thresholds for assigning strains to the same species; they also indicate that isolate MGRD01-02^T^ is most closely related to the *A. durhamensis* and *A. robiniae* strains. Corresponding amino acid identity and conserved protein data not only support these relationships but also confirm the taxonomic integrity of the genus *Actinocrinis*. Based on these results, it is proposed that isolate MGRD01-02^T^ (= CCMM B1308^T^ = ICEBB-09^T^ = NCIMB 15218^T^) be classified in the genus *Actinospica* as *Actinospica acidithermotolerans* sp. nov. The draft genome of the isolate and its closest phylogenomic neighbours contain biosynthetic gene clusters with the potential to produce new natural products, notably antibiotics.

**Supplementary Information:**

The online version contains supplementary material available at 10.1007/s00203-022-03058-7.

## Introduction

Novel filamentous neutrophilic actinomycetes isolated from extreme biomes are a rich source of novel antibiotics (Bull and Goodfellow 2019), as exemplified by the discovery of novel polyketide antibiotics from the type strains of *Micromonospora maris* (Nouioui et al. 2018) and *Streptomyces leeuwenhoekii* (Busarakam et al. 2014) which were isolated from deep-sea sediment and hyper-arid Atacama Desert soil, respectively. Compared with their neutrophilic counterparts, acidophilic filamentous actinomycetes have rarely featured in bioprospecting campaigns even though they are common in acidic habitats and produce diverse specialized metabolites, notably novel antibiotics (Wang and Donk 2012). In general, acid-loving filamentous actinomycetes encompass acidotolerant (pH range 4.5–7.5., optimal growth around pH 5.5) and obligate acidophiles (pH range 3.5–6.5, optimal growth around pH 4.5 (Williams et al. 1971), as represented by *Streptomyces* (Xu et al. 2006) and *Actinospica* species (Cavaletti et al. 2006; Golinska et al. 2015), respectively. Members of these taxa and related genera that contain acidotolerant and acidophilic species are of particular interest as a prospective source of new specialized metabolites as they have large genomes with many biosynthetic gene clusters (BGCs) associated with the production of novel antibiotics (Nouioui et al. 2018; Świecimska et al. 2020) and hence can be considered as gifted sensu Baltz (2017).

The family *Actinospicaceae* (Cavaletti et al. 2006) of the order *Catenulisporales* (Donadio et al. 2015) contains the genera *Actinospica* (Cavaletti et al. 2006) and *Actinocrinis* (Kim et al. 2017); the former encompasses three validly published species, including *Actinospica robiniae*, the nomenclatural type species, and the latter *Actinocrinis puniceicyclus.* The present study, a continuation of our earlier work on the diversity of filamentous actinomycetes from Indonesian extreme habitats, was designed to establish the taxonomic status of an *Actinospica* strain isolated from acidic hot spring sediment. Strain MGRD01-02^T^ was compared with the type strains of *Actinospica, Actinocrinis* and *Catenulispora* species using genomic and phenotypic data. In addition, the draft genomes of the isolate and its closest relatives were checked for natural product-biosynthetic gene clusters (NP-BGCs) predicted to express for novel specialized metabolites, especially antibiotics. The isolate was shown to belong to a new *Actinospica* species: the name proposed for this taxon is *Actinospica acidithermotolerans* with isolate MGRD01-02^T^ as the type strain.

## Materials and methods

### Isolation, maintenance and cultivation

Strain MGRD01-02^T^ was isolated from a composite sediment sample (pH 3.0 ± 0.05, temperature 41.07 ± 0.2 °C, organic matter 0.06 ± 0.02%, salinity 0.03 ± 0.02) collected from the Mengeruda acidic hot spring (8°42′32.224″S/121°5′12.526″E) in East Nusa Tenggara Province, Flores Island, Indonesia. The strain was isolated on acidified actinomycete isolation agar (HiMedia, Mumbai, India), pH 4.5, after 2 weeks at 37 °C following inoculation of the plates with particles of the dried sediment. The pH of the isolation medium and other acidified media were adjusted using KH_2_PO_4_/HCI, KH_2_PO_4_ and KH_2_PO_4_/NaOH buffers. The isolate together with *Actinospica acidiphila* NRRL B-24432^T^. *Actinospica durhamensis* CSCA 57^T^ (Golinska et al. 2015), *Actinospica robiniae* DSM 44927^T^ (Cavaletti et al. 2006), *Actinocrinis puniceicyclus* DSM 45618^T^ and *Catenulispora acidiphila* DSM 44928^T^, were maintained on modified Bennett’s agar at pH 4.5 (Jones 1949) and as a mixture of hyphal fragments and spores in 20% w/v glycerol at − 20 and − 80 °C. The *C. acidiphila* strain was obtained from the collection of the Northern Regional Research Laboratory, Peoria, USA, the *A. durhamensis* strain from the personal collection of Michael Goodfellow (Newcastle University) and the remaining strains from the Leibniz Institute, DSMZ German Collection of Microorganisms and Cell Cultures GmbH, Braunschweig, Germany.

### Chemotaxonomic and morphological properties

Biomass for the chemotaxonomic studies on isolate MGRDO1-02^T^ was prepared in 250 ml of acidified yeast extract-malt extract broth (International *Streptomyces* Project [ISP 2]) (Shirling and Gottlieb 1966) pH 4.5, at 28 °C for 14 days and the resultant biomass harvested by centrifugation at 1968*g* for 10 min, washed twice in sterile distilled water and freeze-dried. The isolate was then examined for diaminopimelic acid isomers, whole cell sugar and polar lipid patterns and its fatty acid and menaquinone profiles determined, in all cases using standard chromatographic procedures and appropriate controls as described previously (Kusuma et al. 2021). The type of cell wall muramic acid was determined after Uchida et al. (1999). In addition, growth taken from an acidified oatmeal agar plate (Küster and Williams 1964), pH 4.5, incubated for 14 days at 28 °C was examined for spore chain arrangement and spore surface ornamentation using a scanning electron microscope (Tescan Vega 3, LMU instrument) in the Electron Microscopy Research Unit, Newcastle University, following the modified procedure described by O’Donnell et al. (1993).

### Phenotypic traits

Smears prepared from growth of isolate MGRD01-02^T^ taken from an acidified oatmeal agar plate after 10 days at 28 °C were examined by light microscopy following Gram staining (Society of American Bacteriologist 1957). The isolate and its phylogenomic neighbours were examined for a broad range of biochemical, degradation and phenotypic properties acquired using methods described by Williams et al. (1983), albeit using acidified media, and for diagnostic enzymes using API-ZYM strips (BioMerieux, Lyon, France). The ability of these strains to grow at different temperature (4, 10, 20, 28, 37, 45 and 55 °C) and pH (4.5–10.5 with increments of 0.5) regimes and in the presence of various sodium chloride concentrations (1, 3 and 5%) was recorded using acidified ISP 2 agar, pH 4.6, as the basal medium. All the tests were carried out in triplicate using a standard inoculum equivalent to 5.0 on the McFarland scale (Murray et al. 1999). Cultural properties of the isolate and its phylogenomic neighbours were recorded on acidified tryptone-yeast extract, yeast extract-malt extract, oatmeal, inorganic salts-starch, glycerol-asparagine, peptone-yeast extract-iron and tyrosine agar plates (ISP media 1–7) (Shirling and Gottlieb 1966) after 21 days at 28 °C. Aerial and substrate mycelial pigment colours and those of diffusible pigments were determined by comparison against colour charts (Kelly 1958).

### Whole-genome sequencing and comparison of sequences

Genomic DNA was extracted from wet biomass of single colonies of isolate MGRD01-02^T^ and *A. durhamensis* CSCA 57^T^ which had been grown on acidified ISP 2 agar (pH 4.5), and on acidified R2A agar (Reasoner and Geldreich 1985), pH 4.5, for *A puniceicyclus* DSM 45618^T^ for 7 days at 28 °C, using the protocol provided by MicrobesNG (Birmingham, UK) (http://www.microbesng.uk), and sequenced on an Miseq instrument (Illumina, San Diego, USA). The quality of DNA preparations and the sequencing of the genomic DNA libraries were carried out following the procedures described by Kusuma et al. (2021). The libraries were sequenced using the 2 × 250 bp paired-end protocol (MicrobesNG), reads under 200 bp discarded and contigs assembled using SPAdes software version 6.1.1 (Bankevich et al. 2012). The draft genomes of the strains were annotated using the RAST-SEED webserver (Aziz et al. 2008) and the default option. Draft genome sequences of isolate MGDR01-02^T^ (GenBank accession number JAGSOH000000000), *A. durhamensis* CSCA 57^T^ (GenBank accession number JAGSOG000000000) and *A. puniceicyclus* DSM 45168^T^ (GenBank accession number JAGSXH000000000) were generated following an established procedure undertaken by MicrobesNG (Birmingham, UK) (http://www.microbesng.uk) and sequenced on an MiSeq instrument (Illumina, San Diego, USA). The quality of the extracted DNA preparations and the sequencing of genomic DNA libraries were achieved as described by Kusuma et al. (2021).

The draft genome sequences generated for the isolate and the, *A. durhamensis* and *A. puniceicyclus* strains were compared with corresponding sequences of *A. acidiphila* NRRL B-24431^T^, *A. robiniae* DSM 44927^T^, and *C. acidiphila* DSM 44928^T^ retrieved from the NCBI genome database using the codon tree option in the PATRIC website (Wattam et al. 2017; Davis et al. 2020), as described by Kusuma et al. (2021), and a ML phylogenomic tree constructed with the RAxML algorithm (Stamatakis 2014). Ortholog average nucleotide identity (orthoANI) (Lee et al. 2016) and digital DNA–DNA hybridization (dDDH) similarities (Meier-Kolthoff et al. 2013a) were determined between all of these organisms using the ANI calculator from the EZBioCloud (https://www.ezbiocloud.net/tools/ani) and the GGDC (http://ggdc.dsm.de/ggdc) webservers, respectively. Corresponding amino acid identity (AAI) (Konstantinidis and Tiedje 2005) and percentage of conserved proteins (POCP) (Qin et al. 2014) were also calculated.

### Phylogeny

An almost full-length 16S rRNA gene sequence (1524 nucleotide [nt], GenBank accession number MK503593.1) was extracted directly from the draft genome of isolate MGRD01-02^T^ using the ContEst16S tool available from the EZBioCloud webserver (https://www.ezbiocloud.net/tools/contest16s). The resultant sequence was found to be identical to one generated using the Sanger method (Sanger and Coulson 1975). The 16S rRNA gene sequences were compared with corresponding sequences of the type strains of *Actinospica*, *Actinocrinis* and *Catenulispora* species taken from the EZBioCloud webserver following multiple sequence alignment using MUSCLE software (Edgar 2010). Pairwise sequence similarities were determined using the single-gene tree option from the Genome-to-Genome Distance Calculator (GGDC) website (Meier-Kolthoff et al. 2013b). Phylogenetic trees were inferred using the maximum-likelihood (ML), maximum-parsimony (MP) and neighbour-joining (NJ) algorithms as previously cited (Golinska et al. 2015) and the trees validated in bootstrap analyses based on 1000 replicates using the MEGA X software package (Kumar et al. 2018), and the GTR + GAMMA model. The trees were rooted using the 16S rRNA gene sequence from *Kineococcus aurantiacus* IFO15268^T^ (GenBank accession number NR_112022.1), the nomenclatural type species of the genus.

### Detection of biosynthetic gene clusters

Natural product BGCs were detected in the draft genomes of isolate MGRD01-02^T^ and its closest phylogenomic neighbours (Table 2) using AntiSMASH 5.0, with default options (Blin et al. 2019), available at http://antismash.secondarymetabolites.org). The genome of the isolate was also screened for the presence of antibiotic resistant target genes using the default settings in the Antibiotic Resistance Target Seeker 2.0 (ARTS 2.0) platform which is designed to detect potential novel antibiotic targets and to prioritize potential new NP-BGCs for further study (Mungan et al. 2020).

## Results and discussion

The morphological and chemotaxonomic properties of the isolate were consistent with its classification in the genus *Actinospica* (Cavaletti et al. 2006; Golinska et al. 2015). The isolate formed an extensively branched substrate mycelium, tufts of white aerial hyphae which differentiated into straight to flexuous chains of cylindrical spores with slightly rugose ornamentation (Fig. S1). Colony characteristics were recorded following growth of the isolate on an oatmeal agar plate after 21 days, as shown in Fig. S2. The diamino acid of the peptidoglycan was 2,6-diamino-3-hydroxydiaminopimelic acid, the muramic acid moieties were N-acetylated, the predominant respiratory quinones consisted of mixtures of hydrogenated menaquinones with nine isoprene units, phosphatidylethanolamine was the diagnostic phospholipid, and the cellular fatty acids were found to be rich in *iso*- and *anteiso*-branched components. These properties distinguish the isolate from species classified in the genera *Actinocrinis* (Kim et al. 2017) and *Catenulispora* (Świecimska et al. 2020).

*Actinospica* species show qualitative differences in sugar and polar lipid patterns, and qualitative and quantitative variations in fatty acid and menaquinone profiles (Cavaletti et al. 2006; Golinska et al. 2015). The major sugars found in whole-organism hydrolysates of isolate MGRD01-02^T^ were galactose, mannose, rhamnose and xylose; the polar lipid pattern contained diphosphatidylglycerol, phosphatidylethanolamine, phosphatidylmethylethanolamine, phosphatidylglycerol and phosphatidylinositol (Fig. S3). These chemotaxonomic properties distinguish the isolate from the type strains of *Actinospica* species, as exemplified by the detection of xylose and phosphatidylglycerol in the sugar and polar lipid profiles, respectively. Like representatives of *A. acidiphila, A. durhamensis* and *A. robiniae* the fatty acid profile of the isolate was composed of major proportions of *iso*-C_15:0_ (25.8%) and *iso*-C_16:0_ (23.7%), but unlike them, it contained major amounts of *anteiso*-C_18:0_/C_18:2_ (33.2%) and only a minor proportion of *anteiso*-C_15:0_. Similarly, the presence of major proportions of di-, tri-, hexa- and octa-hydrogenated menaquinones with nine isoprene units (19, 21, 26 and 29%, respectively) in the isolate distinguishes it from profiles found in the *Actinospica* strains, as illustrated by the presence of a large proportion of MK9 (H_2_). However, quantitative differences in fatty acid and menaquinone profiles need to be interpreted with care as the former are sensitive to growth and experimental conditions (O’Donnell 1988) and the latter by the stage in the growth cycle from which biomass is harvested (Saddler et al. 1986; Yassin et al. 1991).

The accession numbers of the draft genomes are given in Table 1 which also shows that the isolate and *A. durhamensis* CSCA 57^T^ have large genomes, albeit ones lower than those of the type strains of *A. acidiphila* (9.6 Mbp, GenBank accession number NJ-JNYX 0000000), *A. robiniae* (9.9 Mbp, GenBank accession number NZ-AZAN00000000) and *C. acidiphila* (10.5 Mbp, GCA—000024025). However, the digital (d) DNA G + C values of the *Actinospica* strains fall within the narrow range of 70.2 to 72.6%. In contrast, the draft genome size of *A. puniceicyclus* DSM 45618^T^ was relatively low at 6.7 Mbp though its in silico G + C value of 70.5% was just short of that recorded for isolate MGRD01-02^T^.

**Table 1:** Genomic features of isolate MGRD01-02^T^ and the type strains of *Actinospica durhamensis* and *Actinocrinis puniceicyclus*

Genomic features	Isolate MGRD01-02^T^	*A. durhamensis* CSCA 57^T^	*A. puniceicyclus* DSM 45168^T^
Genome range (Mbp)	7.9	9.6	6.7
Coverage	89	73	37
Numbers of:			
Contigs	429	958	434
rRNA genes	4	6	8
tRNA genes	55	60	46
CDS	7811	8999	6330
Contig N_50_	50,301	26,914	45,308
Contig L_50_	47	104	46
Digital DNA G + C (%)	70.6	71.1	70.4
GenBank accession numbers	JAGSOH000000000	JAGSOG000000000	JAGSXH000000000

**Fig. 1:**
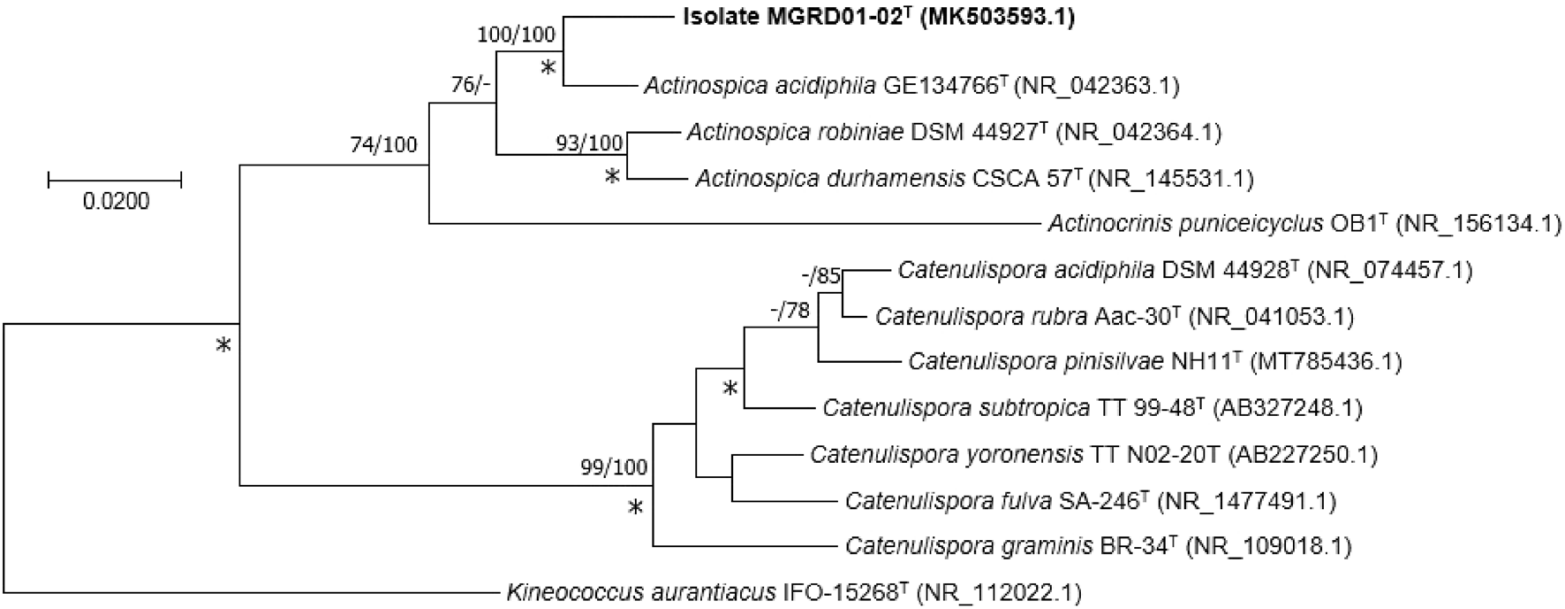
Maximum-likelihood (ML) and maximum-parsimony (MP) trees inferred using the GTR + GAMMA model based on almost complete 16S rRNA gene sequences showing relationships between isolate MGRD01-02^T^ and the type strains of *Actinocrinis, Actinospica* and *Catenulispora* species. Numbers above the nodes indicate bootstrap support values above 60% for the ML (left) and MP (right) algorithms. Asterisks indicate branches recovered using the neighbour-joining algorithm. GenBank accession numbers are shown in parentheses. The scale bar indicates 0.02 substitutions per nucleotide position. The root position of the tree was determined using the type strain of *Kineococcus aurantiaca,* the nomenclatural type species of the genus

The phylogenetic trees (Fig. 1) based on the 16 rRNA gene sequences showed that the isolate, the type strains of the *Actinospica* species and *A. puniceicyclus* DSM 45618^T^ formed a well-supported clade that was most closely related to a similarly well-defined lineage that corresponded to the genus *Catenulispora*. The isolate formed a well-supported branch that was most closely related to *A. acidiphila* NRRL B-24481^T^ sharing a sequence similarity with the latter of 98.4%, a value that corresponded to 22 nt differences at 1446 sites, though this relationship was not supported by a high bootstrap value. These strains shared lower similarity values with the *A. durhamensis* and *A. robiniae* strains and an even lower value of 95.40% with *A. puniceicyclus* DSM 45618^T^, this similarity value is equivalent to 84 nt differences at 1394 locations. The strains assigned to the *Actinospica* 16S rRNA gene clade shared sequence similarities with the *Catenulispora* strains within the range 92.0–92.8%, which is equivalent to 100 to 140 nt differences, respectively. The recovery of the *Actinospica* and *Catenulispora* strains in sister clades reinforces results recorded from earlier studies (Nouioui et al. 2018; Golinska et al. 2015; Świecimska et al. 2020) though in the latter two studies the type strain of *A. puniceicyclus* formed a distinct phyletic line towards the periphery of the *Actinospica* clade.

**Fig. 2:**
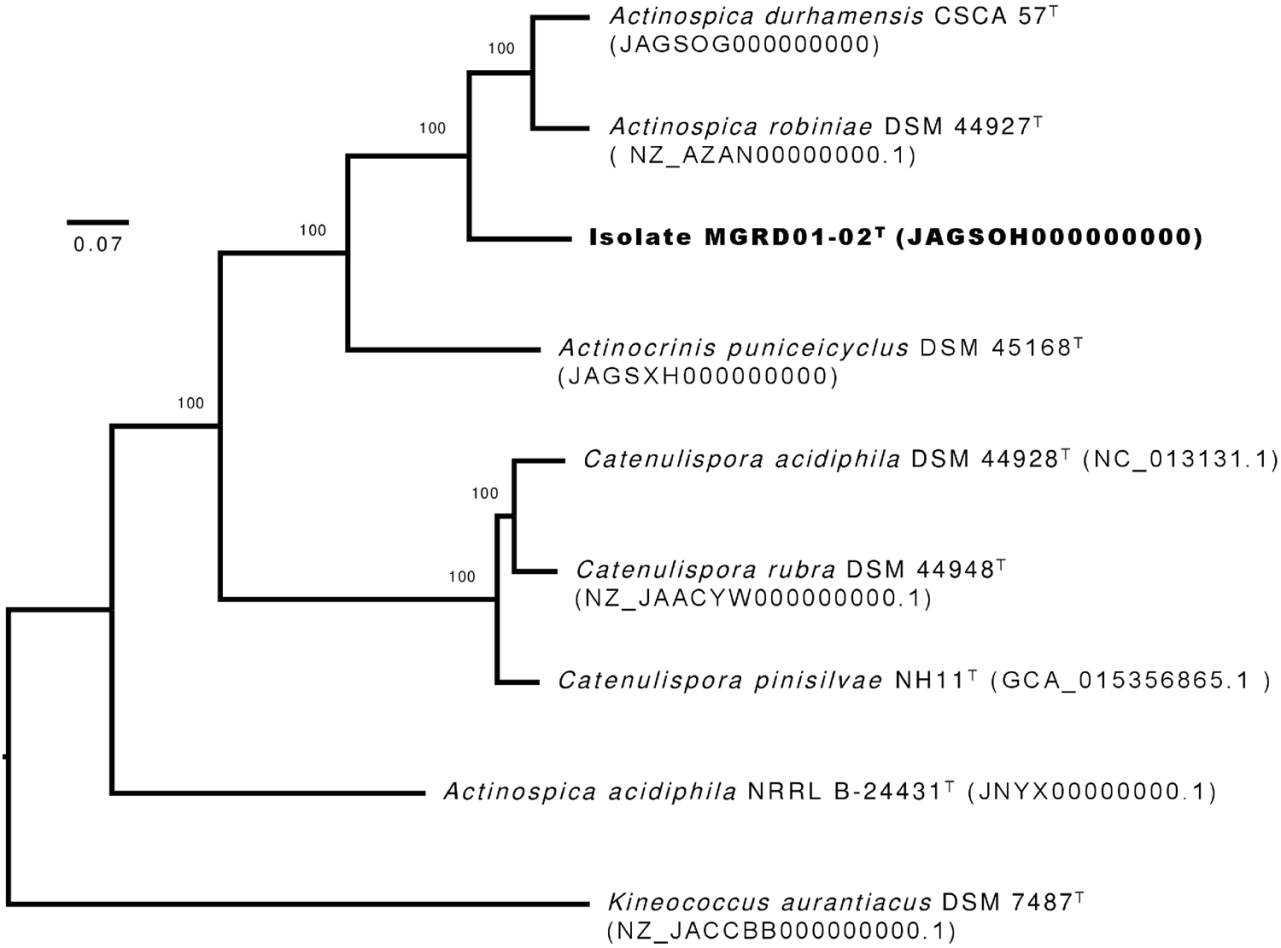
Maximum-likelihood phylogenomic tree based on 441 single copy core genes showing relationships between isolate MGRD01-02^T^ and the type strains of *Actinospica*, *Actinocrinis* and *Catenulispora* species. Numbers at the nodes are bootstrap support values based on 100 replicates calculated using the RAxML Fast Bootstrapping method. GenBank accession numbers are shown in parentheses. The scale bar indicates 0.07 substitutions per nucleotide position. The tree is rooted using the type strain of *Kineococcus aurantiaca,* the nomenclatural type species of the genus

It is evident from the phylogenomic tree (Fig. 2) based on 441 single core genes that isolate MGRD01-02^T^ and its phylogenomic neighbours were recovered as a well-supported clade which corresponded to the order *Catenulisporales* (Donadio et al. 2015). The isolate and the type strains of *A. durhamensis* and *A. robiniae* formed a distinct subclade while those of *A. puniceicyclus, C. acidiphila* and *A. acidiphila* were recovered as well-separated lineages within the tree that were increasingly distant from the subclade. Table 2 shows that all the strains, including the isolate, shared ANI and dDDH similarities much lower than the thresholds (95–96% and 70%, respectively) used to delineate closely related species (Chun et al. 2018). These data also show that the isolate is most closely related to *A. durhamensis* CSCA 57^T^ and *A. robiniae* DSM 44926^T^ and most distantly to *A. acidiphila* NRRL B-24431^T^ and *C. acidiphila* DSM 44928^T^. The *A. puniceicyclus* strain showed ANI and dDDH values with the other strains well below the cut-offs cited above. The close relationship between the isolate, *A. durhamensis* and *A. robiniae* was supported by AAI and POCP values that were well above the 70 and 50% thresholds used to assign species to the same genus (Konstantinidis and Tiedje 2005; Qin et al. 2014), as shown in Table 2. In contrast, the *A. puniceicyclus* and *C. acidiphila* strains shared AAI and POCP similarities well below the recommended thresholds indicating that they belong to different genera. Similarly, the ANI and dDDH values found between the *A. acidiphila* and the other strains is consistent with its assignment to a separate genus though additional studies are needed to confirm this.

**Table 2:** Amino acid and average nucleotide identities, digital DNA:DNA hybridization values and conserved protein similarities between isolate MGRD01-02^T^ and its closest phylogenomic neighbours and between the reference strains

Strains		dDDH/orthoANIu/AAI/POCP values (%)					
		1	2	3	4	5	6
1	Isolate MGRD01-02^T^	–	–	–	–	–	–
2	*Actinospica acidiphila* NRRL B-24431^T^	20.6/72.9/54.8/41.0	–	–	–	–	–
3	*Actinospica durhamensis* CSCA 57^T^	24.1/75.6/72.6/60.8	20.1/72.8/54.6/39.8	–	–	–	–
4	*Actinospica robiniae* DSM 44926^T^	23.6/85.7/71.8/60.0	19.8/72.2/54.4/48.2	29.3/84.7/79.5/67.5	–	–	–
5	*Actinocrinis puniceicyclus* DSM 45168^T^	21.4/75.6/64.2/54.2	19.4/72.6/55.1/38.9	21.7/75.7/63.5/49.2	21.7/75.7/63.5/51.1	–	–
6	*Catenulispora acidiphila* DSM 44928^T^	20.8/74.0/57.2/46.6	19.7/73.9/54.5/41.5	20/73.8/57.3/48.3	20.5/73.9/57.9/49.7	20.9/73.9/57.5/43.3	–

The triplicated cultures gave identical results for all of the phenotypic characteristics shown in Table 3. It is also encouraging that the results for many of the tests confirmed those recorded in earlier analyses on the *Actinospica, Actinocrinis* and *Catenulispora* type strains (Golinska et al. 2015; Świecimska et al. 2020; Kim et al. 2017). All the strains were aerobic, Gram-stain, and catalase positive though other phenotypic features were weighted to distinguish between them, as shown in Table 3. The isolate, for instance, can be separated from all of the other strains as it degraded hypoxanthine and grew at 45*C, and from *A. durhamensis* CSCA 57^T^ and *A. robiniae* DSM 44927^T^, its nearest phylogenomic relatives, by its ability to use inulin as a sole carbon source and by an inability to produce α- and β-galactosidases, β-glucuronidase and cystine, leucine and valine arylamidases. Similarly, the isolate and the other *Actinospica* strains can be separated from the *Actinocrinis* and *Catenulispora* strains as they are positive for esterase (C4), esterase lipase (C8) and β-glucosidase. The *A. acidiphila* strain, unlike the isolate and the *A. durhamensis* and *A. robiniae* strains, degraded starch and used acetamide and L-alanine as sole nitrogen sources. In contrast, all the strains grew optimally at or around pH 5.5 indicating that they are acidotolerant (Williams et al. 1971; Xu et al. 2006).

**Table 3:** Phenotypic characteristics which distinguish isolate MGRD01-02^T^ from its closest phylogenomic neighbours

Characteristics	Strains					
	1	2	3	4	5	6
**API-ZYM tests**						
Acid and alkaline phosphatases, α-glucosidase	+	+	+	+	+	−
α-Chymotrypsin, trypsin	−	−	+	+	+	−
Cystine arylamidase	−	−	+	+	−	−
Esterase (C4), esterase lipase (C8) β-glucosidase	+	+	+	+	−	−
α- and β-Galactosidase, leucine and valine arylamidases	−	+	+	+	+	−
β-Glucuronidase	−	+	+	+	−	−
Lipase (C14)	−	+	+	−	−	−
α-Mannosidase	−	+	−	+	−	−
**Biochemical tests**						
Nitrate reduction	+	+	+	−	+	−
Oxidase	+	+	+	+	+	−
**Degradation tests (% w/v)**						
Hypoxanthine (0.4)	+	−	−	−	−	−
Starch (1)	−	+	−	−	+	+
Tween 20 (1)	−	−	+	−	−	+
Tweens 40 and 60 (1)	+	+	+	+	−	−
Xanthine, xylan (0.4)	−	−	−	−	+	+
**Nutritional tests**						
*Sole carbon sources (1%, w/v)*						
Inulin	+	−	−	−	−	−
d-Mannitol	−	−	−	−	+	−
d-Raffinose	+	+	−	+	+	+
d-Sucrose	−	−	−	−	+	−
d-Trehalose	+	+	+	−	+	+
*Sole nitrogen sources (1%, w/v)*						
Acetamide, l-alanine	−	+	−	−	+	+
l-*Iso*leucine, l-valine	−	+	+	−	−	−
l-Phenylalanine	−	−	+	−	−	+
*Tolerance tests*						
Growth in presence of NaCl (%, w/v)	0–1	0–1	0–1	0	0	1–3
pH range	4.5–6.5	4.5–6.0	4.0–6.0	4.8–6.0	3.5–6.5	6.0– 10.0
Optimal pH Temperature range (°C) Optimal temperature (°C)	5.5 20–45 37	5.0 20–37 28	5.5 10–45 28	5.5 10–37 22–28	5.5 10–45 25	5.0 10–37 22–28

Strains: 1. Isolate MGRD01-02^T^, 2. *Actinospica acidiphila* NRRL B-24431^T^, 3. *Actinospica durhamensis* CSCA 57^T^, 4. *Actinospica robiniae* DSM 44926^T^, 5. *Actinocrinis puniceicyclus* DSM 45168^T^, 6. *Catenulispora acidiphila* DSM 44928^T^. +, positive, −, negative. The strains were positive for naphthol-AS-BI-phosphohydrolase (API-ZYM test), produced was H_2_S and used d-glucose as a sole carbon source, but did not form α-fucosidase (API-ZYM test), reduce nitrite, use acetate, benzoate, fumarate, pyruvate or succinate (sodium salts) as sole carbon sources or degrade adenine (0.5w/v), casein (1), chitin (0.4), elastin (0.3), gelatin (1), guanine (0.3), uric acid (0.4), Tween 80 (1) or l-tyrosine (0.4). The optimal pH and temperatures of the strains ranged from pH 5.0–5.5 and from 25 to 28 °C

As with the phenotypic characteristics shown in Table 3 good congruence was found between the growth and cultural features of the isolate and corresponding results from the previous studies cited previously thereby providing further evidence of the value of cultural properties in the systematics of filamentous actinomycetes (van der Aart et al. 2019). The isolate grew particularly well on oatmeal (Fig. S2) and yeast extract–malt extract agar plates as did the *Actinospic*a and *Catenulispora* strains, but showed varying responses on the remaining ISP media (Table S1). In contrast, *A. puniceicyclus* DSM 45168^T^ did not grow on any of the ISP media. Some of the substrate mycelial pigments were of diagnostic value though this was less so with other colonial features as most of the strains did not produce aerial hyphae or diffusible pigments. However, the isolate can be separated from the other *Actinospica* strains as it formed a greyish-yellow substrate mycelium on yeast extract-malt extract and oatmeal agar plates and a brown diffusible pigment on inorganic salts-starch agar.

### Detection of biosynthetic gene clusters

**Fig. 3:**
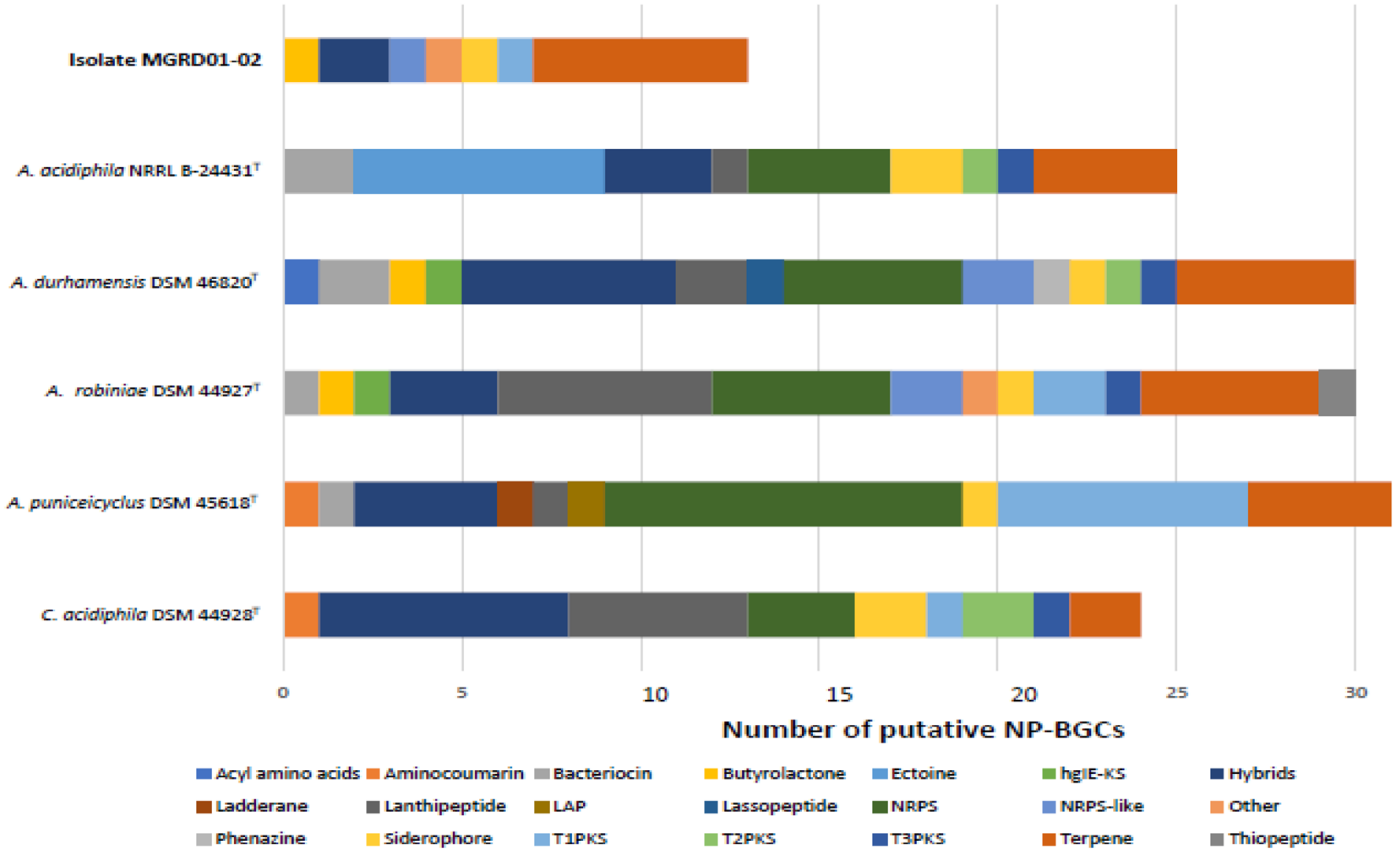
Putative natural product biosynthetic gene clusters (NP-BGCs) detected in the draft genome of isolate MGRD01-02^T^ and in those of the type strains of *Actinospica*, *Actinocrinis* and *Catenulispora* species using the default option of AntiSMASH 5.0

Isolate MGRD01-02^T^ and its closest phylogenomic relatives (Table 2) have large genomes (6.7–10.5 Mbp) which harbor between 13 and 31 BCCs, as shown in Fig. 3. The bioclusters include ones predicted to encode for drug-like molecules, notably non-ribosomal peptide synthases (NRPS), type 1 and 2 polyketide synthases (PKS) and hybrid clusters. The latter are composed of two or more gene clusters and are important as they tend to express for novel derivatives of known compounds (Gallagher and Jensen 2015). Only 5 out of the 66 BCGs predicted to encode for drug-like molecules (7.5%) showed high gene sequence similarities, that is, above 70% with known bioclusters held in the MIBiG database, namely ones associated with the production of antimycin (100% gene identity), cacibiocin B (92% gene identity), catenulipeptin (100% gene identity), curamycin (100% gene identity) and icosalides A/B (100% gene identity), the balance either showed low similarities with known compounds or were predicted to synthesize novel compounds.

The genomes of all of the strains contained a biocluster associated with the production of lobosamide, a polyene macrolactam which inhibits the growth of *Trypanosoma brucei* (Schulze et al. 2015). In contrast, most bioclusters were either strain specific or present in more than one of the genomes. Putative BGCs associated with the production of sporolide were found in the genomes of isolate MGRD01-02^T^, *A. durhamensis* CSCA57^T^ and *A. robiniae* DSM 44926^T^ whereas the biocluster predicted to encode for macrotetralide was only detected in the genomes of the isolate and the *A. pumiceicyclus* strain.

Strain-specific BCCs included ones associated with the production of tiacumicin B (6% gene identity), an anti-bacterial compound (Xiao et al. 2011), atratumycin (21% gene identity), an anti-tubercular antibiotic (Sun et al. 2019), auroramycin (11% gene identity), an antibiotic that actively inhibits *Staphylococcus aureus* (Duggar 2011), catenulipeptin (100% gene identity), a novel class III lantipeptide (Wang and Donk 2012), kirromycin (8% gene identity), an anti-bacterial compound active against *Brevibacterium brevis* (Wolf et al. 1972) and ristomycin A (10% gene identity), an antibiotic which inhibits mycobacteria (Fairbrother 1958) were characteristic of the genomes of isolate MGRD01-02^T^, *A. puniceicyclus* DSM 45618^T^, *A. acidiphila* DSM 44926^T^*, C. acidiphila* DSM 44928^T^, *A. durhamensis* CSCA 57^T^ and *A. robiniae* DSM 44927^T^, respectively. The genome of the isolate also contained a biocluster predicted to encode for feglymycin (10% gene identity), a calcium-dependent antibiotic with anti-HIV properties (Férir et al. 2012). The genome analyses based on The Antibiotic Resistance Target Seeker (ARTS) software version 2.0 highlighted BCG 16.1 which is associated with the production of a feglymycin-like compound (47% gene identity) and hence is a good candidate for further gene expression studies.

The genome mining analyses show that strains classified in the order *Catenulisporales* are a potentially rich source of new specialized metabolites, notably antibiotics. However, molecular studies are needed to determine the functional impact of bioclusters found to predict for unknown products or ones that showed low levels of gene similarity with known compounds. Even so, these studies indicate that *Actinospica* and *Catenulispora* strains should be considered as candidates for bioprospecting campaigns designed to discover novel specialized metabolites of biotechnological value, not least antibiotics with new modes of action.

## Conclusions

This polyphasic study shows that isolate MGRD01-02^T^ is an authentic member of the genus *Actinospica.* Critically, it can be distinguished from the type strains of *Actinospica* species using a combination of genomic, genotypic and phenotypic features. Consequently, it should be classified as a novel species in the genus *Actinospica* for which the name *Actinospica acidithermotolerans* sp. nov. is proposed. The results of this study also confirm the taxonomic integrity of the genus *Actinocrinis* (Kim et al. 2017). It is becoming increasingly apparent that taxonomically diverse actinomycetes, including novel taxa, are a feature of geographically diverse hot springs (Song et al. 2009; Habib et al. 2020). Improved procedures are needed to selectively isolate and characterize novel actinomycetes, including *Actinospica* strains, from hot springs for biotechnological and ecophysiological purposes.

### Description of ***Actinospica acidithermotolerans*** sp. nov.

*Actinospica acidithermotolerans* sp. nov. (a.ci.di.ther.mo.to.’le.rans L. masc. adj. *acidus* sour., Gr. masc. adj. *thermos* hot.,L. pres. part. *tolerans,* tolerating; N.L. part. adj, *acidothermotolerans*, tolerating acid and heat conditions).

Aerobic, Gram-stain positive, actinomycete which forms an extensively branched substrate mycelium, tufts of white aerial hyphae that differentiate into long straight to flexuous chains of cylindrical spores (0.8–0.9 × 0.4–0.5 µm) with slightly rugose ornamentation. Grows from 20 to 45 °C, optimally at ~ 37 °C, from pH 4.5 to 6.5, optimally at ~ pH 5.5 and in the presence of 1% w/v sodium chloride. Grows well on acidified Bennett’s, inorganic salts-starch and starch-casein agar and forms a grayish yellow substrate mycelium and white aerial hyphae on oatmeal agar. Reduces nitrate but not nitrite. Degrades hypoxanthine, Tweens 40 and 60, but not starch, Tween 20, xanthine or xylan. Positive for acid and alkaline phosphatases, esterase (C4), enterase lipase (C8), α- and β- glucosidases, but negative for α-chymotrypsin, cystine, leucine and valine arylamidases, β-glucuronidase, lipase (C14), α- mannosidase and trypsin. d-raffinose and d-trehalose are used as sole carbon sources for energy and growth, but not d-mannitol or sucrose. Does not use acetamide, l-alanine, l-isoleucine, l-phenylalanine or l-valine as sole nitrogen sources. The wall peptidoglycan contains 2,6-diamino-3-hydroxydiaminopimelic acid., N-acetylated muramic acid moieties and galactose, mannose, rhamnose and xylose. The major fatty acids are *iso*-C_15:0_, *iso*-C_16:0,_ and summed feature *anteiso*- C_18:0_/C_18:2_, C_18:0_ is also present (7.2%), the balance of the fatty acids are found in trace amounts (< 0.7%). The polar lipid profile consists of diphosphatidylglycerol, phosphatidylethanolamine, phosphatidylmethylethanolamine, phosphatidylglycerol and phosphatidylinositol and the major menaquinones are MK-9 (H_2_, H_4_, H_6_ and H_8_). The genomic G + C content of the type strain is 70.5% and its approximate genome size 7.9 Mbp.

The type strain, MGRD01-02^T^ (= CCMM B1308^T^ = ICEBB-09^T^ = NCIMB 15218^T^), was isolated from sediment collected from the Mengeruda acidic hotspring in East Nusa Tenggara Province, Flores Island, Indonesia.

## Supplementary Information

Below is the link to the electronic supplementary material.

Supplementary file1 (DOC 512 KB)
