# Supplementary material for: Actinospica acidithermotolerans sp. nov., a novel actinomycete isolated from sediment from an Indonesian hot spring
Source: Arch Microbiol. 2022 Jul 23;204(8):518. doi: 10.1007/s00203-022-03058-7 (PMC9308616; doi:10.1007/s00203-022-03058-7)
Supplement: Supplementary file 1 — Supplementary file1 (DOC 512 KB) [file 203_2022_3058_MOESM1_ESM.doc]

**Supplementary Data**

**Genome-based classification of *Actinospica acidithermotolerans* sp. nov., an actinobacterium isolated from an Indonesian hot spring**

**Ali B. Kusuma, Kurniawan E. Putra, Leggina R. Vanggy, Joshua Loh, Imen Nouioui, Michael Goodfellow**

**
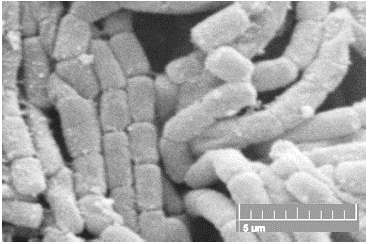
**

**Figure S1**. Scanning electron micrograph of *Actinospica* isolate MGRD01-02^T^ showing straight to flexuous chains of slightly rugose ornamented cylindrical spores following growth on oatmeal agar (pH 4.5) for 14 days at 28^o^C. Bar, 5 µm.


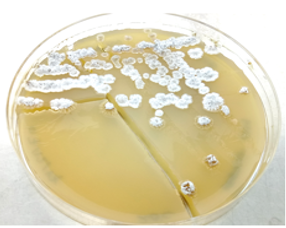


**Figure S2**. Colony morphology of *Actinospica* isolate MGRD01-02^T^ following growth on acidified oatmeal agar pH 4.5 after 21 days incubation at 28^o^C.


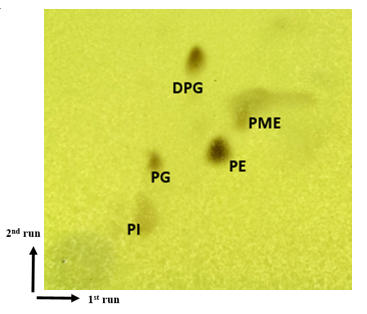


**Figure S3.** Two-dimensional thin-layer chromatography of the polar lipids of isolate MGRD01-02^T^ stained with molybdatophosphoric acid spray (Sigma P1518). Key: DPG, diphosphatidylglycerol; PG, phosphatidylglycerol; PE, phosphatidylethanolamine; PME, phosphatidylmethylethanolamine and PI, phosphatidylinositol., these lipids were identified by comparing their mobilities with those of lipid standards (PH-9; Sigma). Solvent I: chloroform: methanol: distilled water (65:25:4 v/v); solvent 2: chloroform: glacial acetic acid: methanol: distilled water (80:12:15:4 v/v). In the first run the TLC plate was positioned in a perpendicular position towards the solvent direction. In the second run, the position of the TLC plate and solvent were in the same direction.

**Table S1**. Growth and cultural features of isolate MGRD01-02^T^ and its closest phylogenomic neighbours growing on acidified ISP media following incubation for 3 weeks at 28^o^C days

| **Strains** | **Growth and cultural characteristic** | **ISP media** | | | | | | |
| --- | --- | --- | --- | --- | --- | --- | --- | --- |
|  |  | **1** | **2** | **3** | **4** | **5** | **6** | **7** |
| Isolate MGRD01-02^T^ | Growth | + | +++ | +++ | +++ | + | + | ++ |
|  | Aerial hyphae | None | White | White | White | None | None | None |
|  | Substrate mycelium | Trace | Greyish-yellow | Greyish-yellow | Greyish-yellow | Trace | Trace | Trace |
|  | Diffusible pigments | None | None | None | Brown | None | None | None |
| *Actinospica acidiphila* NRRL  B-24431^T^ | Growth | ++ | +++ | +++ | +++ | ++ | + | ++ |
|  | Aerial hyphae | White | White | White | None | White | None | White |
|  | Substrate mycelium | Greyish-yellow | Beige | Cream to greenish | White | Light greenish | White | Greenish |
| *Actinospica durhamensis* CSCA 57^T^ | Growth | ++ | +++ | +++ | +++ | ++ | + | ++ |
|  | Aerial hyphae | None | None | None | None | White | None | None |
|  | Substrate mycelium | Light yellowish brown | Medium yellow | Light yellowish brown | Greenish white | White | Medium yellow | White |
| *Actinospica robiniae* DSM 44926^T^ | Growth | ++ | +++ | +++ | + | + | + | ++ |
|  | Aerial hyphae | None | White | White | None | None | - | None |
|  | Substrate colour | White | Light yellow/ brown | Beige to yellowish | White | White | - | White |
| *Catenulispora acidiphila* DSM 44928^T^ | Growth | ++ | +++ | +++ | +++ | +++ | ++ | +++ |
|  | Aerial hyphae | None | None | White | None | White | None | White |
|  | Substrate mycelium | Yellowish Dark brown | Yellowish Dark brown | Dark reddish Cream to light brown | Cream to light brown | Brown | Yellowish brown | Brown/greyish medium mahogany |
|  | Diffusible pigments | None | Brown | Brownish | Brownish | Light brown | None | Brown |

Key: Growth: +++, abundant; ++, moderate; +, poor; -, no growth.

*Actinocrinis puniceicyclus* DSM 45618^T^ did not grow on any of the ISP media; the type strains of *A. acidiphila, A. durhamensis* and *A. robiniae* did not produced diffusible pigments on any of the media.

ISP media: 1. tryptone-yeast extract., 2. yeast extract-malt extract, 3. oatmeal., 4. inorganic salts-starch., 5. glycerol-asparagine., 6. peptone-yeast extract-iron and 7. tyrosine agar (Shirling and Gottlieb 1966).

**Table S2**. Predicted biosynthetic gene clusters detected in the genomes of the isolate and its closest phylogenomic neighbours

| No | Type | Most similar known cluster | Biosynthetic classes | Percentage similarity | Strains | | | | | |
| --- | --- | --- | --- | --- | --- | --- | --- | --- | --- | --- |
|  |  |  |  |  | Isolate MGRD01-02^T^ | *Actinospica durhamensis*  CSCA 57^T^ | *Actinocrinis puniceicyclus*  DSM 45618^T^ | *Actinospica acidiphila*  NRRL B-24432^T^ | *Actinospica robiniae*  DSM 44927^T^ | *Catenulispora acidiphila*  DSM 44928^T^ |
| 1 | Acyl_amino_acids |  |  |  |  | 1 |  |  |  |  |
| 2 | Aminocoumarin | Cacibiocin B | other | 92% |  |  |  |  |  | 1 |
| 3 | Bacteriocin |  |  |  |  | 1 |  |  |  |  |
| 4 | Bacteriocin | α,β-epoxyketone | nrps-t1pks | 15% |  | 1 |  |  |  |  |
| 5 | Bacteriocin |  |  |  |  |  |  |  | 1 |  |
| 6 | Bacteriocin |  |  |  |  |  |  | 1 |  |  |
| 7 | Baacteriocin | Informatipeptine | RiPP: Lanthipeptide | 57% |  |  |  | 1 |  |  |
| 8 | Bacteriocin PKS-like |  |  |  |  |  | 1 |  |  |  |
| 9 | Betalatone, NRPS | Kutznerides | NRPS | 17% |  |  |  |  | 1 |  |
| 10 | Betalactone, NRPS, T1PKS | Sporolide | nrps-t1pks | 46% |  | 1 |  |  |  |  |
| 11 | Betalactone, T3PKS, NRPS | Streptonigrin | other | 9% |  | 1 |  |  |  |  |
| 12 | Butyrolactone |  |  |  |  | 1 |  |  |  |  |
| 13 | Butyrolactone |  |  |  |  |  |  |  | 1 |  |
| 14 | Butyrolactone |  |  |  | 1 |  |  |  |  |  |
| 15 | Butyrolactone |  |  |  | 1 |  |  |  |  |  |
| 16 | Ectoin | Ectoin | other | 100% |  |  |  | 1 |  |  |
| 17 | hglE-KS |  |  |  |  | 1 |  |  |  |  |
| 18 | hglE-KS | Eicoseicosapentaenoic acid | other | 10% |  |  |  |  | 1 |  |
| 19 | Ladderane | Atratumycin | NRP | 21% |  |  | 1 |  |  |  |
| 20 | Lanthipeptide |  |  |  |  | 1 |  |  |  |  |
| 21 | Lanthipeptide |  |  |  |  | 1 |  |  |  |  |
| 22 | Lanthipeptide |  |  |  |  |  |  |  | 1 |  |
| 23 | Lanthipeptide |  |  |  |  |  |  |  | 1 |  |
| 24 | Lanthipeptide |  |  |  |  |  |  |  | 1 |  |
| 25 | Lanthipeptide | Labyrinthopeptin A1,A3 / labyrinthopeptin A2 | lanthipeptide | 40% |  |  |  |  | 1 |  |
| 26 | Lanthipeptide |  |  |  |  |  |  |  | 1 |  |
| 27 | Lanthipeptide |  |  |  |  |  |  |  | 1 |  |
| 28 | Lanthipeptide |  |  |  |  |  | 1 |  |  |  |
| 29 | Lanthipeptide |  |  |  |  |  |  | 1 |  |  |
| 30 | Lanthipeptide |  |  |  |  |  |  |  |  | 1 |
| 31 | Lanthipeptide |  |  |  |  |  |  |  |  | 1 |
| 32 | Lanthipeptide |  |  |  |  |  |  |  |  | 1 |
| 33 | Lanthipeptide | Catenulipeptin | RiPP: Lanthipeptide | 100% |  |  |  |  |  | 1 |
| 34 | Lanthipeptide |  |  |  |  |  |  |  |  | 1 |
| 35 | LAP |  |  |  |  |  | 1 |  |  |  |
| 36 | LAP, thiopeptide | Meridamycin | NRP + polyketide | 7% |  |  |  |  |  | 1 |
| 37 | LAP, thiopeptide |  |  |  |  |  |  |  |  | 1 |
| 38 | Lassopeptide | Paulomycin | other | 3% |  | 1 |  |  |  |  |
| 39 | Lipolanthine, lanthipeptide |  |  |  |  |  |  |  |  | 1 |
| 40 | NRPS |  |  |  |  | 1 |  |  |  |  |
| 41 | NRPS | Ristomycin A | nrps-saccharide | 10% |  | 1 |  |  |  |  |
| 42 | NRPS | Cahuitamycins | NRPS | 12% |  | 1 |  |  |  |  |
| 43 | NRPS | Lobosamide | t1pks | 6% |  | 1 |  |  |  |  |
| 44 | NRPS |  |  |  |  | 1 |  |  |  |  |
| 45 | NRPS |  |  |  |  |  |  |  | 1 |  |
| 46 | NRPS | Labosamide | t1pks | 8% |  |  |  |  | 1 |  |
| 47 | NRPS | Labosamide | t1pks | 10% |  |  |  |  | 1 |  |
| 48 | NRPS | Phthoxazolin | nrps-t1pks | 4% |  |  |  |  | 1 |  |
| 49 | NRPS | Azicemicin | t2pks | 4% |  |  |  |  | 1 |  |
| 50 | NRPS | Chloramphenicol | NRP | 23% |  |  | 1 |  |  |  |
| 51 | NRPS |  |  |  |  |  | 1 |  |  |  |
| 52 | NRPS | Lobosamide A / Lobosamide B / Lobosamide C | polyketide | 25% |  |  | 1 |  |  |  |
| 53 | NRPS | Erythrochelin | NRP |  |  |  | 1 |  |  |  |
| 54 | NRPS | Sceliphrolactam | polyketide | 8% |  |  | 1 |  |  |  |
| 55 | NRPS |  |  |  |  |  | 1 |  |  |  |
| 56 | NRPS | Icosalide A / Icosalide B | NRP: Lipopeptide | 100% |  |  | 1 |  |  |  |
| 57 | NRPS |  |  |  |  |  | 1 |  |  |  |
| 58 | NRPS |  |  |  |  |  | 1 |  |  |  |
| 59 | NRPS |  |  |  |  |  | 1 |  |  |  |
| 60 | NRPS | SCO-2138 | RiPP | 64% |  |  |  | 1 |  |  |
| 61 | NRPS | Auroramycin | polyketide | 11% |  |  |  | 1 |  |  |
| 62 | NRPS | Sarpeptin A / Sarpeptin B | NRP | 41% |  |  |  | 1 |  |  |
| 63 | NRPS | Marformycin A / Marformycin B / Marformycin C / Marformycin D / Marformycin E / Marformycin F | NRP | 12% |  |  |  | 1 |  |  |
| 64 | NRPS | Lobosamide A / Lobosamide B / Lobosamide C | polyketide | 8% |  |  |  |  |  | 1 |
| 65 | NRPS | Crochelin A | NRP + polyketide | 11% |  |  |  |  |  | 1 |
| 66 | NRPS | s56-p1 | NRP | 39% |  |  |  |  |  | 1 |
| 67 | NRPS, Bacteriocin | Daptomycin | NRPS | 6% |  |  | 1 |  |  |  |
| 68 | NRPS, hgIE-KS, T1PKS | Simocyclinone D8 | Saccharide + Polyketide: Modular type I + Polyketide: Type II + Other: Aminocoumarin |  |  |  |  |  |  | 1 |
| 69 | NRPS, Lanthipeptide | Calcium-dependent antibiotic | NRPS | 10% | 1 |  |  |  |  |  |
| 70 | NRPS, T3PKS, other | Feglymycin | NRPS | 47% | 1 |  |  |  |  |  |
| 71 | NRPS, T1PKS | Kirromycin | nrps-t1pks+transatpks | 8% |  |  |  |  | 1 |  |
| 72 | NRPS, T1PKS | BE-43547A1 / BE-43547A2 / BE-43547B1 / BE-43547B2 / BE-43547B3 / BE-43547C1 / BE-43547C2 | NRP: Cyclic depsipeptide + Polyketide: Modular type I | 30% |  |  | 1 |  |  |  |
| 73 | NRPS, T1PKS | Antimycin | NRP + polyketide | 100% |  |  |  | 1 |  |  |
| 74 | NRPS, transAT-PKS, T1PKS, PKS-like | Guangnanmycin | NRP + polyketide | 35% |  |  |  |  |  | 1 |
| 75 | NRPS-like |  |  |  |  | 1 |  |  |  |  |
| 76 | NRPS-like | Feglymycin | nrps-t1pks | 26% |  | 1 |  |  |  |  |
| 77 | NRPS-like | Vazabitide A | NRPS | 4% |  |  |  |  | 1 |  |
| 78 | NRPS-like | Octacosamicin | nrps-t1pks | 8% |  |  |  |  | 1 |  |
| 79 | NRPS-like |  |  |  | 1 |  |  |  |  |  |
| 80 | NRPS-like, bacteriocin, terpene | 2-methylisoborneol | terpene | 100% |  |  |  |  |  | 1 |
| 81 | NRPS-like, PKS-like | LL-D49194α1 (LLD) | polyketide | 3% |  |  |  | 1 |  |  |
| 82 | NRPS-like, T1PKS |  |  |  |  | 1 |  |  |  |  |
| 83 | NRPS-like, Phosphonate |  |  |  |  |  | 1 |  |  |  |
| 84 | Other |  |  |  |  |  |  |  | 1 |  |
| 85 | Other |  |  |  | 1 |  |  |  |  |  |
| 86 | Other |  |  |  | 1 |  |  |  |  |  |
| 87 | Phenazine | Lomofungin | other | 69% |  | 1 |  |  |  |  |
| 88 | PKS-like, Butyrolactone | Macrotetrolide | polyketide | 25% |  |  | 1 |  |  |  |
| 89 | Siderophore |  |  |  |  | 1 |  |  |  |  |
| 90 | Siderophore |  |  |  |  |  |  |  | 1 |  |
| 91 | Siderophore |  |  |  |  |  | 1 |  |  |  |
| 92 | Siderophore | Grincamycin | polyketide: Type II + Saccharide: Hybrid/tailoring | 8% |  |  |  | 1 |  |  |
| 93 | Siderophore | Desferrioxamin B / Desferrioxamine E | other | 83% |  |  |  | 1 |  |  |
| 94 | Siderophore |  |  |  |  |  |  |  |  | 1 |
| 95 | Siderophore | Desferrioxamin B | other | 100% |  |  |  |  |  | 1 |
| 96 | Siderophore | Macrotetrolide | t2pks | 33% | 1 |  |  |  |  |  |
| 97 | T1PKS |  |  |  |  |  |  |  | 1 |  |
| 98 | T1PKS | Sporolide | nrps-t1pks | 12% |  |  |  |  | 1 |  |
| 99 | T1PKS | Meoabyssomicin / Abyssomicin | polyketide | 25% |  |  | 1 |  |  |  |
| 100 | T1PKS | Azalomycin F3a | polyketide | 34% |  |  | 1 |  |  |  |
| 101 | T1PKS | JBIR-100 | polyketide: modular type I | 38% |  |  | 1 |  |  |  |
| 102 | T1PKS | Marineosin A / Marineosin B | polyketide | 9% |  |  | 1 |  |  |  |
| 103 | T1PKS | Salinomycin | polyketide: modular type I | 10% |  |  | 1 |  |  |  |
| 104 | T1PKS |  |  |  |  |  | 1 |  |  |  |
| 105 | T1PKS |  |  |  |  |  | 1 |  |  |  |
| 106 | T1PKS | Amycolamycin A / Amycolamycin B | polyketide | 10% |  |  |  |  |  | 1 |
| 107 | T1PKS | Tiacumicin B | t1pks | 6% | 1 |  |  |  |  |  |
| 108 | T1PKS, NRPS | Butyrolactol | t1pks | 33% |  | 1 |  |  |  |  |
| 109 | T1PKS, NRPS | Nanchangmycin | Polyketide | 9% |  |  |  |  |  | 1 |
| 110 | T2PKS | Curamycin | t2pks | 100% |  | 1 |  |  |  |  |
| 111 | T2PKS | Spore pigment | polyketide | 83% |  |  |  | 1 |  |  |
| 112 | T2PKS | Spore pigment | polyketide | 66% |  |  |  |  |  | 1 |
| 113 | T2PKS | Prejadomycin / rabelomycin / gaudimycin C / gaudimycin D / UWM^ / gaudimycin A | Polyketide: Type II + Saccharide: Hybrid/tailoring | 27% |  |  |  |  |  | 1 |
| 114 | T2PKS | Actinorhodin | Poluketide: type II | 45% |  |  |  |  |  | 1 |
| 115 | T2PKS, PKS-like, Betalactone | LL-D49194α1 (LLD) | polyketide | 45% |  |  |  | 1 |  |  |
| 116 | T2PKS, NRPS | Spore pigment | t2pks | 75% |  |  |  |  | 1 |  |
| 117 | T3PKS | Balhimycin | NRPS | 10% |  | 1 |  |  |  |  |
| 118 | T3PKS | Tylactone | polyketide | 6% |  |  |  |  | 1 |  |
| 119 | T3PKS | Lakylresorcinol | polyketide | 100% |  |  |  | 1 |  |  |
| 120 | T3PKS | Acarviostatin I03 / Acarviostatin II03 / Acarviostatin III03 / Acarviostatin IV03 | saccharide | 25% |  |  |  |  |  | 1 |
| 121 | Terpene | SF2575 | t2pks-saccharide-other | 6% |  | 1 |  |  |  |  |
| 122 | Terpene | Hopene | terpene | 38% |  | 1 |  |  | 1 | 1 |
| 123 | Terpene | 2-methylisoborneol | terpene | 75% | 1 | 1 |  |  | 1 |  |
| 124 | Terpene | Geosmin | terpene | 100% | 1 | 1 |  | 1 | 1 | 1 |
| 125 | Terpene | Thiolutin | NRPS | 8% |  | 1 |  |  |  |  |
| 126 | Terpene |  |  |  |  |  |  |  | 1 |  |
| 127 | Terpene | SF2575 | t2pks-saccharide-other | 8% |  |  |  |  | 1 |  |
| 128 | Terpene | SF2575 | polyketide: Type II + Saccharide: Hybrid/tailoring | 4% |  |  | 1 |  |  |  |
| 129 | Terpene | Hopene | terpene | 30% |  |  | 1 |  |  |  |
| 130 | Terpene |  |  |  |  |  | 1 |  |  |  |
| 131 | Terpene | Pentalenolactone | terpene | 29% |  |  | 1 |  |  |  |
| 132 | Terpene | Albaflavenone | terpene | 100% |  |  |  | 1 |  |  |
| 133 | Terpene | Caratenoid | terpene | 54% |  |  |  | 1 |  |  |
| 134 | Terpene | Hopene | terpene | 61% |  |  |  | 1 |  |  |
| 135 | Terpene |  |  |  | 1 |  |  |  |  |  |
| 136 | Terpene | Hopene | terpene | 46% | 1 |  |  |  |  |  |
| 137 | Terpene |  |  |  | 1 |  |  |  |  |  |
| 138 | Terpene |  |  |  | 1 |  |  |  |  |  |
| 139 | Terpene |  |  |  | 1 |  |  |  |  |  |
| 140 | Thiopeptide |  |  |  |  |  |  |  | 1 |  |
| 141 | Thiopeptide, LAP | Feglymycin | NRPS | 10% |  | 1 |  |  |  |  |
| 142 | Thiopeptide, LAP | Lactozole | thiopeptide | 44% |  | 1 |  |  |  |  |
